# Applying immunity and metabolism to psychiatry: lost, or in translation?

**DOI:** 10.1192/bjp.2026.10714

**Published:** 2026-07-14

**Authors:** Riccardo De Giorgi, Margaret K Hahn, Thomas Cheliotis-James, Emily CC Smith, Brenda WJH Penninx, Toby Pillinger, Adam Al-Diwani

**Affiliations:** 1 Department of Psychiatry, University of Oxford, Oxford, UK; 2 NIHR Oxford Health BRC, Oxford Health NHS Foundation Trust, Oxford, UK; 3 Department of Experimental Medicine, University of Salento, Lecce, Italy; 4 Centre for Addiction and Mental Health (CAMH), Toronto, Canada; 5 Institute of Medical Science, University of Toronto, Toronto, Canada; 6 Department of Pharmacology & Toxicology, University of Toronto, Toronto, Canada; 7 Department of Psychiatry, University of Toronto, Toronto, Canada; 8 Banting and Best Diabetes Centre, University of Toronto, Toronto, Canada; 9 Department of Clinical Medicine, Faculty of Health and Medical Sciences, University of Copenhagen, Copenhagen, Denmark; 10 Department of Psychosis Studies, Institute of Psychiatry, Psychology & Neuroscience, King’s College London, London, UK; 11 NIHR Maudsley BRC, South London and Maudsley NHS FT, London, UK; 12 TUNE-UP Service, South London and Maudsley NHS Foundation Trust, London, UK; 13 Department of Psychiatry, Amsterdam UMC, Vrije Universiteit Amsterdam, Amsterdam, The Netherlands

## Abstract

Immunologic, metabolic, and increasingly ‘immuno-metabolic’ approaches, are prominent in contemporary psychiatric discourse, yet translation into clinical practice remains variable. In this Feature, we pragmatically ask where signal ends and speculation begins, and what this means for the clinical psychiatrist.

Autoimmune encephalitis provides a rare but instructive yardstick in which antibody-based mechanisms map onto distinct neuropsychiatric syndromes and respond to targeted immunotherapy. However, while the broader concept of autoimmune psychosis may extend beyond this tightly defined niche, it remains largely experimental and requires stronger evidence.

More broadly applicable concepts, including an immuno-metabolic subtype of depression, are increasingly supported by mechanistic work, with attendant treatment implications, although differentiation from routine holistic care remains less clear. In psychosis and severe mental illness, immune and cardiometabolic dysfunction may contribute to both psychiatric and physical disease burden beyond lifestyle or treatment effects alone. Emerging therapies, including GLP-1-based approaches, may bind these threads together with gains for body and mind, but specific evaluation within psychiatry continues.

Overall, our view is that the opportunities are not lost, but for robust translation, there is an ongoing need for precise, incremental research, inter-disciplinary collaboration and rigorous communication of nuance.

## Introduction

Immune, metabolic, or overlapping ‘immuno-metabolic’ mechanisms are garnering much attention in academic psychiatry and popular science. Establishing causal relevance and interpreting research data for clinical reality, however, remains incomplete. While a multitude of data teaches us that psychiatric disorders are heterogeneous in cause, presentation, and management, there is always a temptation to expect unitary causes and treatments. Here, we seek to survey the extent to which immune and/or metabolic approaches to psychiatry are challenging or affirming a hope-hype-disillusionment cycle. In this non-exhaustive approach, we use examples with which we, as active researchers in the field are familiar to give a flavour of the translational process. We seek to be a ‘critical friend’, both giving a platform to current and future robust translation, but questioning the extent to which these concepts have and will move beyond the conference stage and into the clinic.

## Neuronal surface autoantibodies – autoimmune encephalitis, and beyond?

Autoimmune encephalitis (AE), a disease driven by autoantibodies against specific neuronal-surface signalling proteins that manifests with neuropsychiatric features via the disruption of brain networks (1, 2). This offers clear bio-reductionist causality and has opened the door to disease-modifying immunotherapies, with symptomatic treatments taking a supportive role. There is now a whole sub-specialty of ‘autoimmune neurology’, so it may be asked where is psychiatry (3)? The direct relationship between antibody, neural target, and symptomatic expression can serve as a yardstick when considering the causal role of immunity in general psychiatric conditions and implies several caveats potentially limiting impact on mainstream psychiatry. The first is that while AE is not as uncommon as sometimes thought – indeed at an incidence of 0.5–1 per 100,000 persons per year it is comparable to infective encephalitis (1) – but this is eclipsed by the prevalence of severe mental illness (SMI) and psychiatric disorders in general. The second is that, while psychiatric and neuropsychiatric clinical features are near-ubiquitous in one of its most common forms, NMDAR-antibody encephalitis, the symptom profile is almost invariably complexed with traditional ‘neurological’ features such as seizures, movement disorders, and/or reduced consciousness, amongst others (4). The third is that, while these conditions can be confused with primary forms of SMI, on closer inspection the psychopathology is a poor fit for the symptomatology of SMI – indeed the combination of abrupt onset and atypicality may help inform diagnostic suspicion (5). For NMDAR-antibody encephalitis, evidence suggests that ‘isolated psychiatric’ presentations are rare, except in relapse after an established poly-symptomatic initial episode (6). Thus, AE is undoubtedly important to know about due to major medical risks of missing the condition, but also the risk of imprecise and potentially excessive investigation in unlikely cases.

It is argued though that restricting the relevance of neuronal-surface autoantibodies in psychiatry to classical AE misses a potentially larger group of patients defined by aspects of the core AE clinical syndrome and a multi-modal set of investigations suggestive of relevant autoimmunity – the ‘autoimmune psychosis’ concept (7). These criteria aim to detect any psychosis that may benefit from immunotherapy, including AE, but also hitherto putatively undescribed conditions. Within this, proponents envisage a role for a form of parallel autoimmune psychiatrist, utilising a similar range of immune treatments to modify disease course of SMI with evidence of autoimmune change sub-clinical for encephalitis but relevant to mechanism (8). There remains considerable discussion, urging caution both conceptually and clinically but equally not wishing to deprive a sizeable group of patients with a novel treatment approach and causal understanding (9, 10).

This discourse has been active since the initial conceptualisation of AE and its clinical and para-clinical limits. The SINAPPS2 trial, where patients with psychosis and serum-only neuronal surface antibodies are being randomised to immunotherapy or placebo in addition to their usual psychiatric care, is expected to report soon (11). This will provide important high-quality evidence towards this question. Overall, we would propose that this broad body of investigation has led to clinically-meaningful and scientifically high-yield insight into interactions between the brain, immunity and clinical neuropsychiatry.

## Disentangling heterogeneity across depressive disorders: the case for an ‘immuno-metabolic’ phenotype of depression

But what of inflammatory mediators such as cytokines more generally in common mental disorders? One driver of interest in immuno-psychiatry has been the success of biological therapies in modifying disease and thereby clinical outcomes in undisputed inflammatory conditions such as rheumatoid arthritis (12). There are also clear examples where inflammatory mediators drive altered mental states relevant to psychiatric disorder, such as the overlap between ‘sickness behaviours’ in viral illness and depressive psychopathology, as well as interferon treatments precipitating depressive symptoms (13). Furthermore, depressive disorders are associated with higher prevalence of endocrine and cardiometabolic comorbidities including cardiovascular disease, obesity, and diabetes (14). These conditions appear linked by dysregulation of immune pathways including systemic low-grade inflammation with elevated levels of C-reactive protein, cytokines, and glycoprotein acetyls, as well as dysregulation of the hypothalamus-pituitary-adrenal (HPA) stress axis, along with metabolic abnormalities involving increased waist circumference, dyslipidaemia, and insulin- and leptin-resistance (15).

This is potentially the most impactful application of immunology to psychiatry: accounts of both sub-groups of common conditions in which inflammation is highly relevant to mental state, such as ‘inflamed depression’ (16), and large contributions to transdiagnostic endophenotypes such as anhedonia and anergia. As data accrues, these concepts are becoming increasingly coherent, nonetheless, these too are still largely in the domain of specialist academic clinics, rather than mainstream psychiatric practice. However, there is also clear and substantial heterogeneity across people with depressive illnesses, as only 25-35% of these individuals present with objective evidence of inflammation and/or metabolic abnormalities (17). This subpopulation could arguably be considered as an ‘immuno-metabolic’ subtype of depression, as they oftentimes display a differential phenotype with higher levels of atypical energy-related symptoms such as hypersomnia, leaden paralysis, fatigue, hyperphagia, and profound anhedonia. Neurobiological studies in this specific subgroup confirm reduced effort-based decision-making, disruption in reward and motor-based brain circuitry, decreased monoamine homeostasis with reduced striatal dopamine availability, and activation of the kynurenine pathway – all of which are correlated with lower motivation and energy levels. Nonetheless, clinicians will need to show caution since this phenotype could also signify bipolar depression, with resultant bipolar disorder-specific management required – emphasising the ongoing importance of clinical history taking despite progress in biomarker science.

If such an immuno-metabolic depression phenotype was systematically assessed and ascertained, more precise and thus effective interventions could be implemented – especially since these persons seem to respond less well to conventional antidepressant therapies (15). The rationale here is to discern higher effect sizes in smaller subgroups. Treatments that directly target inflammation, insulin-resistance, or lipid imbalances could therefore be useful complementary interventions. Evidence that anti-inflammatory (e.g., celecoxib or infliximab) or lipid-lowering (e.g., statins) medications could contribute to depressive symptom reduction has thus far been inconclusive. The concept should though not be rejected out of hand since, as newer efforts are pointing out, patients tended to not be grouped on the basis of immuno-metabolic biomarkers (18). It is hoped that a precision medicine approach will more fairly evaluate the approach. Equally important in view of their baseline elevated risk for cardiometabolic morbidity and mortality, patients with immuno-metabolic depression could particularly benefit from in-depth medical monitoring and management of their physical health. Lifestyle interventions such as physical exercise and running therapy, behavioural activation, smoking cessation, and sleep hygiene regimens could help further reduce depressive symptoms while also improving the overall health status – although, plausibly, these strategies would specifically benefit patients across the depressive spectrum and health promotion in general (19).

Ultimately, more research is still needed to help establish robust clinical and neurobiological criteria to identify those with depressive syndrome phenotypes who could then benefit from these specific interventions. Efforts such as the ‘advanced stratification of people with depression based on inflammation’ (ASPIRE) initiative are actively testing this model for precision immuno-psychiatry (20). Depression remains a highly heterogeneous category and whether immuno-metabolic depression as a specific subtype survives further scrutiny remains open to future investigation. Indeed, if the broader immuno-metabolic framework outlined above is to be rigorously evaluated and meaningfully advanced, integration of additional metabolic biomarkers will be required to develop a sufficiently robust and clinically-informative model.

## Heart health in psychosis: a further paradigm of the immuno-metabolic approach?

In the field of heart health, an intriguing hypothesis seeks to integrate immunological and cardiometabolic dysregulation into the fundamental biology of SMI. While there is clearly uncertainty, longitudinal cohort evidence from the Avon Longitudinal Study of Parents and Children (ALSPAC) cohort suggests an interaction between inflammatory and metabolic factors linked to increased risk of psychotic experience (21). One interpretation that challenges the traditional attribution of excess mortality solely to lifestyle and medication side-effects is whether there are shared biological pathways predisposing to both cardiac and psychiatric dysfunction. Such reframing not only offers new insights into disease mechanisms, but also suggests the possibility of earlier, biologically-informed interventions targeting a potentially unifying pathophysiology to complement traditional psychiatric bio-psycho-social strategies.

People with psychosis die around 15-20 years earlier than the general population, with heart disease as the leading cause of excess mortality (22). Growing evidence indicates that psychosis itself is intrinsically linked to physiological changes that predispose to heart failure. Even before antipsychotic treatment, people with first-episode psychosis show systemic metabolic dysregulation and inflammation (23). Mendelian randomisation studies demonstrate that genetically-predicted schizophrenia increases heart failure risk (24). Higher schizophrenia genetic liability is also associated with impaired diastolic relaxation, reflected by reduced absolute peak diastolic strain rates (25). Diastolic dysfunction is a well-established precursor of heart failure, providing a plausible pathway by which genetic risk for schizophrenia contributes to increased heart failure risk, with immune pathways likewise implicated in this relationship. Cardiac magnetic resonance imaging further illustrates that apparently healthy individuals with schizophrenia exhibit myocardial inflammation and fibrosis compared with controls (26). If psychosis is intrinsically linked to immune and metabolic pathways driving cardiac dysfunction, this opens the door to novel interventions. Beyond lifestyle modification and metabolic side-effect management, mechanistic therapies that directly target inflammation and bioenergetics hold promise. For example, GLP-1 therapies not only promote weight loss and improve glycaemic control but also exert cardioprotective effects (27), while anti-inflammatory agents may attenuate immune-driven cardiac remodelling (28).

To move from mechanistic insight to clinical benefit, several steps are required. First, large-scale longitudinal cohorts with deep phenotyping are needed to disentangle causal pathways linking psychosis to cardiac dysfunction. Second, early-phase trials should test whether targeting these pathways improves intermediate cardiac outcomes such as diastolic function and myocardial inflammation. Third, longer-term studies are needed to establish whether such improvements translate into reduced progression to clinical heart failure and premature mortality in people with psychosis. Parallel work should also explore personalised approaches, identifying which patients carry the greatest biological risk and may derive most benefit.

## The role of the psychiatrist as advocate and coordinating figure for metabolic monitoring and care in people with mental illness: an ongoing call for action

While novel therapeutics are being developed, it is essential to act on what we already know. People with schizophrenia continue to receive substandard cardiovascular care. Ensuring gold-standard monitoring and management of cardiovascular health in schizophrenia is an immediate and achievable priority. Improved implementation of existing guidelines could reduce premature mortality even before new immuno-metabolic treatments are realised (29).

**Figure 1:**
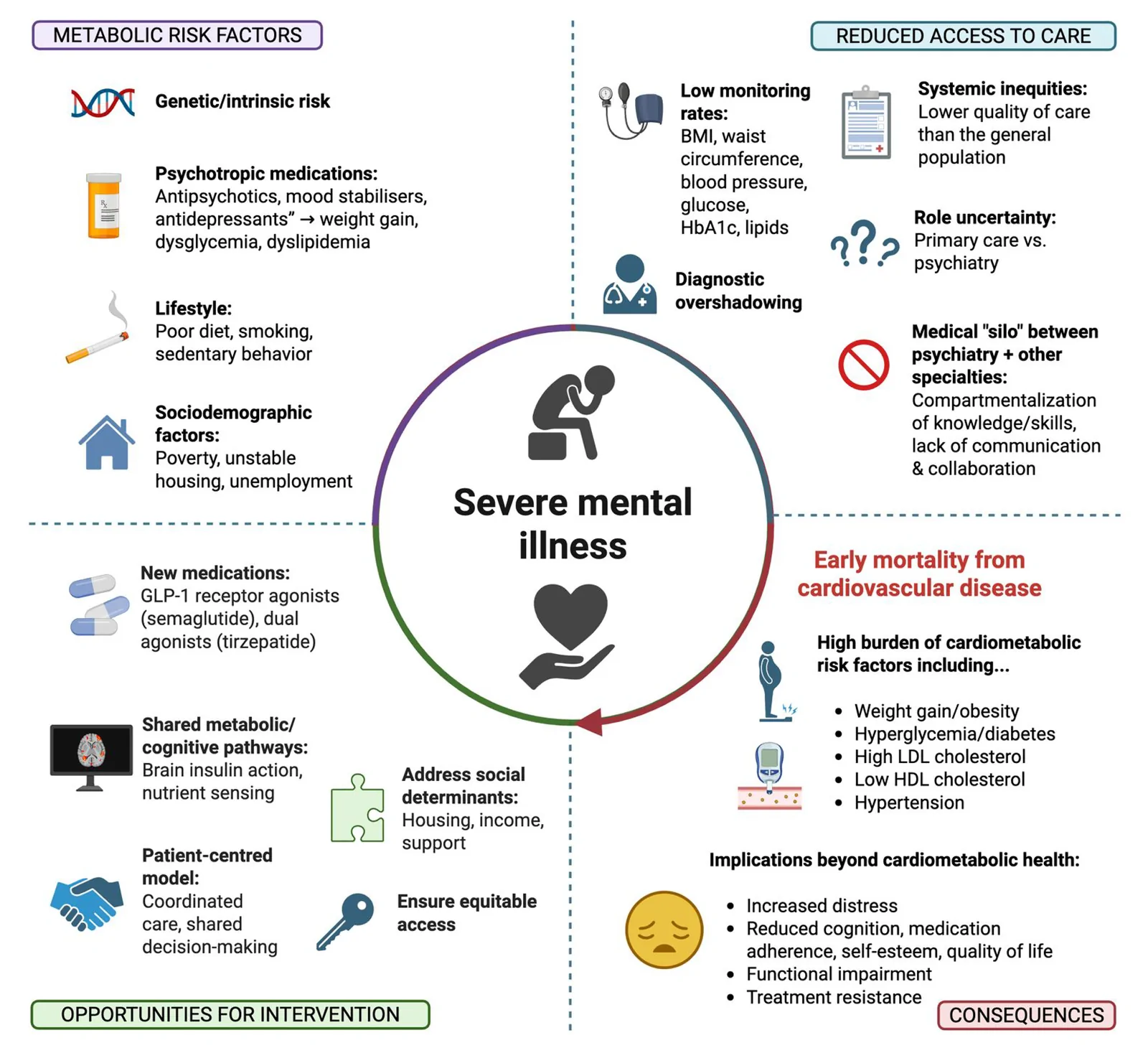
Putative unique immune and bioenergetic pathways to cardiometabolic disease in people with psychosis

Metabolic risk factors in SMI are more complex than in the general population and linked to genetics, illness-related lifestyle factors, socioeconomic characteristics, and reduced access to medical care (Figure 1). Moreover, SMI is complicated by psychotropic treatments. While antipsychotics remain life-saving medications, they can also cause weight gain, dysglycaemia, and dyslipidaemia, particularly in younger, treatment-naïve individuals (22). Similarly, mood stabilisers and many antidepressants also cause clinically-relevant cardiometabolic disturbances after only a few weeks of treatment (30). In addition to exacerbating intrinsic metabolic risk in patients with SMI, these side effects can present barriers to social engagement and psychological well-being by impairing medication adherence and self-esteem, and by worsening pre-existing cognitive dysfunction (31). Wherever possible, psychiatrists should prioritise treatments with lower metabolic liability; for instance, recent consensus guidelines recommend aripiprazole as a first-line agent in people experiencing a first episode of psychosis (29).

It has been shown that individuals with comorbid mental and physical illness experience a lower quality of care than those with a physical illness alone (14). Indeed, disparities in care have been hypothesised to contribute to the increases in mortality gap from cardiovascular disease in SMI as compared to the general population, where declines in morality have been observed. Routine monitoring of body mass index, waist circumference, blood pressure, haemoglobin A1c, glucose, and lipids is recommended across existing guidelines to ensure that metabolic abnormalities can be promptly identified and treated, yet notably uptake remains poor or sporadic (32). Interventions to prevent and target modifiable cardiometabolic risk factors remain under-prescribed and under-utilised. These glaring gaps in care likely stem from a combination of individual and structural factors including diagnostic overshadowing, limited resources, and role uncertainty between psychiatrists and primary care providers. The INTEGRATE consensus guidance also clarifies that psychiatrists should play a central and proactive role in the detection, monitoring, and management of metabolic disturbance in people with SMI (29).

There remains a case to be made for ‘liaison medicine’, helping to consolidate metabolic expertise within mental health services, while developing integrated cross-service pathways would ensure that abnormalities identified through screening lead to timely evidence-based care. Importantly, this is not about delegation of responsibility away from psychiatry, which remains central, but about confirming that specialist input is available to ensure patients with SMI receive the most up-to-date medical care, without putting undue burden beyond already stretched core activities. Similar to the value of liaison psychiatry in bringing psychiatric expertise into the general hospital, including upskilling hospital practitioners, the liaison physician could add value in other direction, engaging with the diverse contexts in which psychiatry is practiced (33).

## Could GLP-1-based therapies become an exemplary immuno-metabolic approach across cognitive and mental disorders?

Beyond adequate physical health monitoring and care and the undeniable value of psychosocial and lifestyle interventions, a recurring theme in our prior examples involves recalibrating the immunological and/or metabolic disturbances that underpin both mental and physical morbidity. While we must caution once more against reductionism and the unwarranted need for a unifying theme that explains the whole complexity of mental illnesses, the – necessarily tentative, at this stage – paradigm upon which we pivot is GLP-1 receptor agonism. GLP-1-based therapies e.g., liraglutide, semaglutide, and tirzepatide, initially developed for diabetes and obesity, have rapidly become blockbuster drugs also owing to their robust effects on cardiovascular outcomes and mortality (27). Their emergence has taken the field of neuropsychiatric research by storm. By addressing the cardiometabolic excess risk that is both intrinsic to severe mental illness and iatrogenic from psychotropic treatment, these medications may provide an impactful solution to ongoing efforts to narrow the mortality gap seen in SMI – so that co-prescribing with antipsychotics that have a high cardiometabolic burden would be potentially an easy win (34).

Perhaps more intriguingly, but also less conclusively, the neuropsychiatric story of these compounds could extend beyond these somewhat obvious somatic benefits. GLP-1 therapies modulate primarily metabolic and neuroendocrine circuits, but also inflammatory pathways implicated in immuno-metabolic phenotypes spanning multiple psychiatric conditions (35–37). GLP-1 receptors are expressed on neurons, astrocytes, and microglia, and their agonism modulates synaptic plasticity, dampens pro-inflammatory cytokines, and preserves blood-brain barrier (BBB) integrity – mechanisms central to neuropathology (36). Large-scale proteomic studies indicate that GLP-1 therapies alter circulating proteins influencing glycaemic, lipid, and inflammatory pathways, independent of weight change (38). These signatures overlap with disease proteomes, such as depression and substance misuse, generating rational repurposing hypotheses. These medications thus offer a model example of how metabolic and immunological – especially inflammatory mechanisms may converge across a spectrum of cognitive and mental illnesses.

Emerging evidence further suggests that GLP-1 therapies can influence neurocognitive and psychological processes through central actions on energy, reward, and other neuropsychological circuits, with translational work actively mapping GLP-1 therapies’ actions across relevant neurobiological circuits (36). Observational studies link GLP-1 therapies with reduced risk of substance use, psychosis, and neurocognitive disorders. Cognition, as a shared vulnerability across dementia and severe mental illness, is a clear target (37). Human evidence remains mixed but biologically plausible, with several studies emphasising the need for biomarker-enriched trials (39). In schizophrenia, semaglutide improved metabolic outcomes but did not affect psychopathology (34), while a recent small but robust clinical trial in 72 people with major depression has shown evidence of overall cognitive benefit (40). To clarify any putative effects on these symptom domains, non-conventional pathways – including microbiota and gut-brain mechanisms, need to be gradually embedded in psychiatric clinical research cohorts. On neuropsychiatric safety, evidence from RCTs and observational cohorts is reassuring, with no consistent increase in psychiatric adverse events or suicidality (41). Nonetheless, we should test risk where it actually sits, for example, people with current mood disorders and a history of suicidal behaviour.

At a disciplinary level, the vivacity of the scientific debate surrounding GLP-1 therapies may also invite self-reflection. Psychiatry has often imported concepts from other specialties; now, it stands at a juncture where it might reciprocate by offering an integrated framework in which cognition, mood, behaviour, immunity, and metabolism are treated not as separate domains, but as interdependent dimensions of a single biological continuum. A bold example that might have concrete real-world consequence would be the automatic inclusion of liaison psychiatry in MDT care for obesity. By tuning reward and impulsivity, GLP-1 therapies may reframe obesity as a condition with tractable cognitive-affective mechanisms rather than a ‘purely somatic’ disorder. Clinically, such perspective supports integrated care, pairing pharmacology with psychosocial interventions to improve weight-loss maintenance. This could also materialise in liaison psychiatrists being actively involved in screening for, and assertively treat mental health consequence from frequent rebound weight gain after stopping medications.

Overall, GLP-1 therapies may signal a future for psychiatry in which mechanism-informed therapies transcend traditional diagnostic and indeed specialty boundaries. However, their full potential will only be realised through rigorous, biomarker-enriched trials capable of disentangling peripheral from central effects, metabolic correction from genuine neuropsychiatric change, and clinical stratification therein. Such studies should prioritise mechanistic endpoints (e.g., cognitive biomarkers, inflammation, brain connectivity) alongside clinical outcomes to delineate the pathways through which these agents exert benefit. For enthusiastic clinicians, however, a note of caution. Current GLP-1 therapies are unlikely to become a neuropsychiatric panacea. They carry both familiar adverse effects (e.g. nausea and other gastrointestinal disturbances, tolerable in the short term but potentially problematic with prolonged or lifelong use, as is often required in psychiatric disorders) and also more significant and less desirable consequences (e.g., weight loss in frail or underweight patients such as those with dementia). Newer or developing compounds acting on related pathways (e.g. glucagon, gastrointestinal peptide or GIP, amylin) will require further characterisation from a neuropsychiatric perspective as they may carry unexpected benefits – or harms. Cost and accessibility are likely to remain major barriers to uptake of these medications, especially in a population traditionally underserved as the one we psychiatrists manage. The more optimistic vision involves precision therapies that harness GLP-1 pathways to target cognition, reward, and inflammation with minimal metabolic trade-off – something that is now being articulated in conceptual frameworks for metabolic psychiatry (42).

## Conclusion

Immunology and metabolism are mature basic biomedical sciences that have come and gone over the years in psychiatric theory and practice (43). The current metabolic wave is coming after several years of focus on low-grade systemic inflammation itself following pioneering observations about links between mind, brain and immunoendocrine systems. It is often held up as a spur to progress that some of our most utilised approaches from all three bio-psycho-social spheres date back decades. Maybe it reflects the effect sizes of these often-serendipitous insights that future treatments would be even better – but the lesson of other waves of ever more precise bio-psychiatry such as genetics and brain imaging is of ever-increasing complexity (44). Similarly, beyond some initial very high effect size examples such as autoimmune encephalitis and the neuropsychiatric impact of interferon treatments, it has taken years to gradually integrate immune and inflammatory insights into psychiatric concepts and begin to leverage powerful multi-omics technologies. GLP-1 therapies are a major focus but after years of careful laboratory and early translational work. While investigators are encouraged to name their sub-field, there is not necessarily a unifying cohesive theory that binds immunology, metabolism, and their overlaps to psychiatry, and indeed one may question why there would need to be. Do we talk of neurochemical psychiatry when thinking of our most commonly prescribed psychotropics? That’s just psychiatry. Overall, our glass is more full than empty – it is a sign of a maturing niche to refine concepts with the accrual of data while embedding clinically-actionable data – not lost, and with far more translation to come.
